# Barriers and facilitators for self-harm prevention and surveillance: A cross-sectional study with primary health care nurses in Brazil

**DOI:** 10.1371/journal.pone.0338182

**Published:** 2025-12-11

**Authors:** Amanda Sarah Vanzela, Laysa Fernanda Silva Pedrollo, Aline Conceição Silva, Isabela dos Santos Martin, Bruno Pereira da Silva, Maria Aline do Nascimento Brandão dos Santos, Jeferson Rodrigues, Márcia Astrês Fernandes, Ana Carolina Guidorizzi Zanetti, Bianca Cristina Ciccone Giacon, Guilherme Oliveira de Arruda, Kelly Graziani Giacchero Vedana

**Affiliations:** 1 Department of Psychiatric Nursing and Human Sciences, School of Nursing, University of São Paulo, Ribeirão Preto, São Paulo, Brazil; 2 Department of Maternal-Child and Psychiatric Nursing, School of Nursing, University of São Paulo, São Paulo, Brazil; 3 Multidisciplinary Centre, Federal University of Acre, Cruzeiro do Sul, Acre, Brazil; 4 Department of Psychology, Federal University of Santa Catarina, Florianópolis, Santa Catarina, Brazil; 5 Department of Nursing, Federal University of Piauí, Teresina, Piauí, Brazil; 6 Integrated Health Institute, Federal University of Mato Grosso do Sul, Campo Grande, Mato Grosso Do Sul, Brazil; Jawaharlal Institute of Postgraduate Medical Education and Research, INDIA

## Abstract

Most individuals who die by suicide do not have contact with specialised mental health services; instead, they engage with primary health care services. This highlights the critical role of primary care in preventing self-harm and suicide. The objective of this study was to identify the barriers, facilitators, and associated factors influencing suicide and self-harm prevention and surveillance from the perspective of primary care nurses. This cross-sectional observational study used a quantitative approach and included 125 nurses from the five macro-regions of Brazil. Data concerning barriers and facilitators to self-harm prevention and surveillance, along with associated factors, were collected online using a data capture software. Instruments were based on the Theoretical Domains Framework, developed and validated for the Brazilian context. Data analysis was performed using Generalised Additive Models for Location, Scale, and Shape (GAMLSS), with distribution selection based on the Akaike Information Criterion (AIC) and the Generalised AIC (GAIC). Analyses were conducted in R (version 4.4.1) with a significance level of 5%. In terms of self-harm prevention, “knowledge and skills” was identified as a facilitator (58.1%), while “context” (75.7%) and “reinforcement” (91.7%) were recognised as barriers. For self-harm surveillance, “reinforcement” (89.8%) and “intentions” (56.5%) were identified as barriers, whereas facilitators included “knowledge and skills” (61.7%), “beliefs about consequences” (82.9%), “emotions” (55.8%), and “behavioural regulation” (61.4%). Identifying barriers and facilitators within healthcare is crucial for implementing evidence-based practices. The findings of this study can contribute to the development of more tailored self-harm prevention strategies across different regions of Brazil.

## Introduction

People who died by suicide often visit Primary Health Care (PHC) services before death [1,2]. Such visits are more frequent in the five years preceding death, intensifying in the last three months before death [3]. Nurses are a vital component of the PHC workforce and are often the first point of contact for patients, thereby playing a crucial role in identifying risk factors and collaborating on self-harm prevention efforts [4]. This highlights the significance of PHC and its workforce in developing and implementing strategies to prevent self-harm [1,5].

PHC services represent the foundation level of a healthcare system, acting as the initial and most accessible point of contact for the population. PHC facilitates health promotion, treatment, rehabilitation, and harm prevention [1,5,6]. Consequently, it is strategic for the early management of both protective and risk factors concerning self-harm and suicide prevention [1,7].

For the purpose of this study, self-harm encompasses self-inflicted injuries, including suicidal behaviours (attempts and deaths by suicide), but excluding stereotypical behaviour [8]. These are considered significant public health concerns in Brazil and globally. In 2021, over 15,000 individuals died by suicide in Brazil, making it the 27th leading cause of death in the country, predominantly affecting young adults and adolescents. Despite the rising incidence of self-harm in the Americas and low and middle-income countries, studies regarding prevention strategies have been mainly focused on high-income countries [1].

In Brazil, cases of self-harm are monitored through two distinct surveillance systems. For suicide-related deaths, death certificates completed by health units are collected and recorded in the Mortality Information System (SIM) [9]. For cases of suicide attempts or self-harm, reporting is conducted through the Notifiable Diseases Information System (SINAN), where reporting is compulsory and must be submitted by the professional who saw the victim within 24 hours of the event [9–10]. Both systems are typically updated monthly in a public-domain national database, although the frequency may vary by state and municipality. Surveillance is vital for comprehending the phenomenon and formulating public policies, making it an essential strategy for self-harm prevention [9].

Other effective self-harm prevention interventions can be implemented in PHC services. These include professionals’ education and training, early identification of risk situations, safety planning, and management of depressive symptoms [11–12]. Studies have shown that interprofessional collaboration, clear communication, effective leadership, and enhancements to electronic systems are facilitators for evidence-based self-harm prevention practices [13].

Nonetheless, previous studies have identified significant barriers to the effective implementation of self-harm prevention interventions [14–16] and emphasised the need for improved self-harm prevention in primary care [16]. Key barriers include understanding the local context, stakeholder involvement, financial resources, human resource training, multisectoral collaboration, data collection, and stigma [11], as well as conflicts between immediate demands and time constraints [13]. Studies in Brazil have highlighted the lack of training and negative attitudes among primary care professionals [18–19]. Recognising the characteristics and scope of these barriers in specific contexts can support the design and monitoring of effective self-harm prevention strategies [11,15,20].

To develop feasible, evidence-based practices that can be effectively implemented in real-world settings, it is essential to identify the barriers and facilitators that may influence their design and implementation. This understanding enables a contextual analysis and planning of strategies that are genuinely relevant to a given reality [15]. When barriers to implementing a practice are poorly understood, it becomes increasingly challenging to engage professionals in its adoption [15]. According to the WHO, it is vital to understand the problems and interventions that are meaningful in a specific context, as barriers to implementation can jeopardise a practice’s success and sustainability [11–21].

To our knowledge, no studies have investigated the barriers and facilitators for self-harm prevention and surveillance in Brazil. Therefore, this study used a Likert-scale survey to identify these barriers, facilitators, and associated factors from the perspective of PHC nurses in Brazil.

## Method

### Design

This is a cross-sectional observational study with a quantitative approach. The results are reported following the Strengthening the Reporting of Observational Studies in Epidemiology (STROBE) guidelines [22].

### Setting

Brazil is a vast, diverse country, divided into five macro-regions (North, Northeast, Central-West, Southeast, and South), with a total population of approximately 212 million inhabitants. Each macro-region comprises micro-regions known as municipalities. These municipalities are grouped into states that operate under federal and state governments but retain the authority to allocate and utilise healthcare funds based on their population-specific needs. This variation in funding usage may influence the organisation and delivery of PHC services, explaining differences in resource availability, accessibility, and policies across regions.

PHC covers the entire Brazilian territory, with nursing professionals constituting the majority of its workforce [23]. In Brazil, PHC encompasses a range of healthcare strategies and services, with the Family Health Strategy (ESF, or *Estratégia de Saúde da Família* in Portuguese) as its primary component. The ESF is central to Brazil’s PHC, comprising multidisciplinary teams that provide consultations, examinations, prevention programmes, and vaccinations. It operates closely with the community and serves as the initial point of contact for Brazilians with the Unified Health System (SUS, or *Sistema Único de Saúde*, in Portuguese).

Mental health (MH) care in Brazil is organised through the Psychosocial Care Network (RAPS), a population-based system extending from PHC to tertiary and emergency levels [24]. One of the key mental health care strategies developed by the Brazilian government is ‘matrix support’. Matrix support is a partnership between PHC and MH services, aimed at enhancing the quality of MH care within the ESF through co-responsibility and interdisciplinary collaboration. The joint efforts between the ESF and specialised MH professionals facilitate accessible MH care in the community, particularly in regions where specialised services may be limited [25–27].

This study was conducted multicentrically, allowing collaboration from researchers from the five macro-regions of the country. Consequently, the participating nurses were recruited from the Southeast, North, Northeast, South, and Central-West regions.

### Participants

Participants were primarily invited and recruited through six posts published on the research group’s Instagram and LinkedIn accounts, which were also shared within the authors’ professional networks. Additionally, the study was advertised twice on the Brazilian Federal Nursing Council’s website and disseminated to at least one university in each of the country’s five macro-regions. Inclusion criteria consisted of being over 18 years of age, holding a higher education degree in nursing, being professionally affiliated with a PHC unit, and having internet access.

The sample size was determined using stratified random sampling with proportional allocation across five strata, representing cities with the highest suicide rates in each of Brazil’s five regions. The sample size was calculated based on the average coverage rates of the Family Health Strategy (ESF) and PHC in each municipality, utilising a 5% relative sampling error and a 5% significance level. A population correction was applied when the calculated sample exceeded 10% of the total population of PHC nurses (N = 2,075). The sample was then allocated proportionally across the strata based on the number of professionals in each city. Although the required sample size was established, recruitment challenges led to a final sample that was smaller than anticipated. This limitation is acknowledged and considered in the interpretation of the results.

### Measures

Data collection instruments addressing barriers and facilitators for self-harm prevention (BFP) and barriers and facilitators for self-harm surveillance (BFS) were developed based on the Theoretical Domains Framework and validated within the Brazilian context [28–29]. The BFP instrument explores professionals’ perceptions regarding factors influencing self-harm prevention in primary care. It includes 11 Likert-scale items, grouped into three domains: knowledge and skills, context, and institutional support (reinforcement). These domains reflect the individual, environmental, and organisational influences that might promote or hinder preventive actions in PHC settings [28].

The BFS assesses professionals’ perceptions of the factors that facilitate or obstruct the reporting and surveillance of self-harm. It includes 16 Likert-scale items across six domains: knowledge and skills, reinforcement, intentions, beliefs about consequences, emotions, and behavioural regulation [29]. Scores for each instrument are calculated by domain. Within each domain, item responses were summed and divided by the number of items, yielding average scores. A higher score (closer to five) indicates a greater perceived presence of facilitators, while a lower score (closer to one) indicates perceived barriers ^(28-29)^.

**Fig 1:**
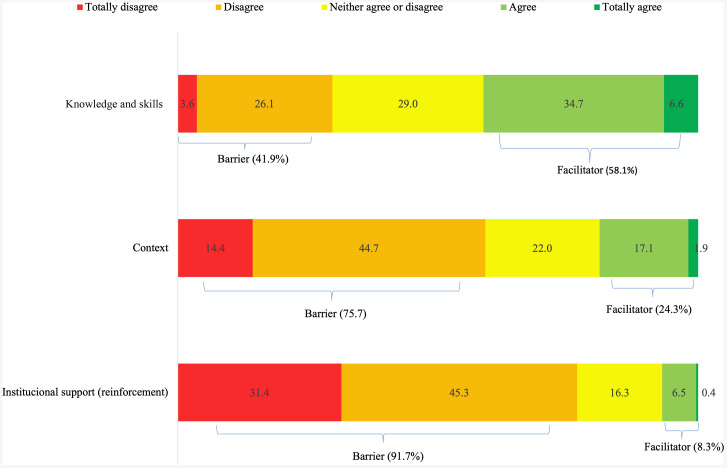
Barriers and facilitators for self-harm prevention according to participants’ responses (n = 125). **(A)** Domain 1 – Knowledge and skills (items: I have the technical skills to carry out self-harm prevention strategy; I know how to carry out self-harm prevention strategy; I feel confident and capable of engaging in suicide prevention strategies; I feel confident in working with victims of self-harm at my workplace; I seek ways to improve my performance to contribute to self-harm prevention in primary care; I know which services in the network [health care and other social devices] I should liaise to work on self-harm prevention), **(B)** Domain 2 – Context (items: My workplace has physical and material resources to carry out the self-harm prevention strategy; The health services available in the network where I work are sufficient to meet demands related to self-harm; My workplace has human resources to carry out a self-harm prevention strategy), **(C)** Domain 3 – Institutional support [reinforcement] (items: At my workplace, there is some goal/indicator related to self-harm prevention; At my workplace, there is a protocol for self-harm prevention).

**Fig 2:**
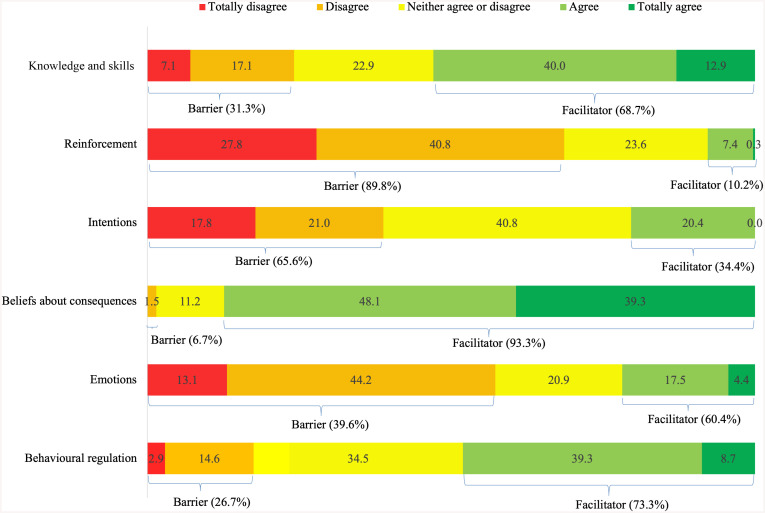
Barriers and facilitators for self-harm surveillance according to primary care nurses (n = 103). **(A)** Domain 1 – Knowledge and skills (items: I am prepared to complete the self-harm surveillance form; I have sufficient experience to carry out self-harm surveillance; I feel confident and capable of carrying out the surveillance; I know how to complete the surveillance form), **(B)**Domain 2 – Reinforcement (items: I am evaluated on my performance in completing the self-harm surveillance form; I am recognised/valued when I complete the self-harm surveillance form; At my workplace, there is some goal, protocol, or routine for notifying self-harm), **(C)**Domain 3 – Intentions (items: I am determined to complete the form whenever I encounter a case of self-harm; I understand the reasons for notifying a case of self-harm; I have more important things to do than complete the self-harm surveillance form), **(D)**Domain 4 – Beliefs about consequences (items: Notification is helpful in preventing self-harm; I expect that notifying self-harm can bring benefits), **(E)**Domain 5 – Emotions (items: Completing the self-harm surveillance form causes me emotional exhaustion; Notifying self-harm triggers anxiety, fear, sadness, or other unpleasant feelings), **(F)**Domain 6 – Behavioural regulation (items: I reflect on my performance in completing the self-harm surveillance form; I seek new ways to improve my performance in completing the self-harm surveillance form).

To enhance clarity and facilitate the comprehensible presentation of the descriptive analysis of results, responses “strongly disagree” and “disagree” were grouped as barriers, while “agree” and “strongly agree” were considered facilitators (Figs 1 and 2). The values for each category were summed and averaged within each domain. Responses labelled as “neither agree nor disagree” were considered neutral and therefore excluded from this classification.

To characterise the sample, demographic and professional data were collected, including region of the country, state, race/colour, age, sex, gender identity, education level, previous training in the topic of self-harm, number of employment positions, time working in PHC, personal and professional experience with self-harm, prior work with victims of self-harm, presence of a specific mental health agenda in the service, presence of matrix support in the service, experience/knowledge about the self-harm surveillance form, existence of local strategy and/or protocol.

### Variables

The outcome variables considered for the analyses were the respective domains of the BFP (knowledge and skills, context, and institutional support) and BFS (knowledge and skills, reinforcement, intentions, beliefs about consequences, emotions, and emotional regulation).

Independent variables related to the BFP domains included: city of employment, race (white/yellow, black/brown); gender (male, female); years since graduation; previous training in the topic of self-harm (yes, no); multiple income streams (more than one); time working in PHC (years); presence of formal mental health initiative/agenda at the workplace (yes, no/don’t know); workplace liaises with matrix support (yes, no/don’t know); lived experiences –self, family, or friends (yes, no); has worked with victims of suicide or self-harm (yes, no); has participated in suicide prevention actions (yes, no); city of employment has a local plan for suicide/self-harm prevention (yes, no/don’t know); workplace has a standard operating procedure related to preventing suicide or self-harm (yes, no/don’t know); and workplace has a specific mental health-related agenda (yes, no/don’t know).

For the BFS-related outcomes, in addition to the independent variables mentioned above, “knowledge about the surveillance form” (yes, no) and “has completed the self-harm surveillance form previously” (yes, no) were also included in the analysis.

### Data collection

Data were collected virtually using a self-administered questionnaire in RedCAP (Research Electronic Data Capture) from May to October 2024. Participants provided informed consent electronically in writing on the data collection software before participating. An optional emailed copy of the consent form was available for review.

A total of 196 nurses expressed interest in the study and completed the questionnaire. To ensure data quality, responses were screened for duplication (defined as multiple submissions from the same participant; n = 10) and for completeness, excluding cases where more than 15% of the questionnaires were left unanswered (n = 61). The final sample comprised 125 PHC nurses from all five regions of Brazil. All participants completed the first instrument related to self-harm prevention (n = 125), and a subset completed the second instrument on self-harm surveillance (n = 103). The surveillance instrument was placed later in the survey for all participants, which might explain differences in completion rates.

### Data analysis

ASV and LSFP had access to the raw data on November 1, 2024. The data were anonymised before analysis to ensure privacy and compliance with ethical standards and then exported to a Microsoft Excel spreadsheet. Missing data in the respective outcomes were excluded to ensure the integrity of the analysis. The Generalised Additive Models for Location, Scale and Shape (GAMLSS) class was used because it allows selection of distributions and statistical models adaptable to the nature of our data, enabling us to capture nuances in distribution patterns and accommodate features such as skewness and variability.

Considering the distributional characteristics of the data, for the domain 1 - “knowledge and skills” (both prevention and surveillance instruments), we used the Logistic (LO) and Box-Cox-Cole-Green (BCCG) distributions. We applied a logarithmic transformation for the remaining domains, where the outcome variables were expressed as proportions or percentages. This helped stabilise variance and normalise skewed distributions, improving model fit and interpretability. As a result, the domain one results are reported as point values, while the other domains are presented as percentages.

To determine the most appropriate combination of response distributions and predictor variables, model selection was guided by the Akaike Information Criterion (AIC) and the Bayesian Information Criterion (BIC), with a penalty value of k = 4, as suggested by Bastiani [30]. Model adequacy was assessed using the Shapiro-Wilk normality test, with a significance level of 5% (α = 0.005). All analyses were conducted using R software (version 4.4.1).

### Strategies to address potential bias

While recruitment via social media may introduce selection bias and limit generalisation, strategies were employed to enhance representativeness and minimise potential bias.

Firstly, standardised data collection was used across all study sites to reduce measurement bias and ensure comparability between groups. Secondly, eligible nurses were selected regardless of their background or experience, and they participated voluntarily and anonymously in the study, which may have reduced social desirability bias in their responses. Thirdly, in the analysis phase, model selection was guided by objective criteria (AIC, GAIC) and all models were adjusted for relevant sociodemographic and professional covariates, reducing confounding bias. Also, the Shapiro-Wilk test was applied to assess model adequacy and identify any potential model outliers that could bias the results.

### Ethical considerations

The study was approved by the Research Ethics Committee of the Ribeirão Preto School of Nursing (CEP/EERP-USP) under protocol number 6.308.903. The research followed the recommendations of CNS Resolution 466/2012 for research with humans, respecting ethical principles and ensuring participant confidentiality.

## Results

From the 125 participants who composed the final sample, the majority were female (91%), from the southern region of Brazil (43%), had a postgraduate degree (62.7%), and had an average of 9.6 years of experience working in PHC (SD = 7.6). Additionally, 68.5% of participants reported lived experiences, and 84.6% reported having worked with a victim of suicide/self-harm, while only 28% had specific self-harm prevention training. Regarding participants’ service characteristics, most services did not have a specific mental health agenda (52%), were not involved in matrix support actions (66.1%), and did not have a local plan for preventing self-harm/suicide (85.5%).

The domain with the highest score related to suicide prevention was “knowledge and skills” (mean = 3.14, SD = 0.68), while “institutional support (reinforcement)” (mean = 1.99, SD = 0.77) and “context” (mean = 2.47, SD = 0.75) presented the lowest scores. Regarding barriers and facilitators related to surveillance, the highest mean scores were obtained in “beliefs about consequences” (mean = 4.25, SD = 0.62) and “intentions” (mean = 4.09, SD = 0.62), while the lowest mean scores were related to “reinforcement” (mean = 2.11, SD = 0.73) (Table 1).

**Table 1:** Domain scores for the “barriers and facilitators for self-harm prevention” and “barriers and facilitators for self-harm surveillance” instruments (n = 125).

Domains	n	mean	sd	min	Q.25	Q.5	Q.75	max
**Prevention**								
1- knowledge and skills	125	3.14	0.68	1.33	2.67	3.17	3.50	5.00
2- context	125	2.47	0.75	1.00	2.00	2.33	3.00	4.67
3- institutional support	125	1.99	0.77	1.00	1.50	2.00	2.50	4.50
**Surveillance**								
1- knowledge and skills	103	3.34	1.04	1.00	2.75	3.50	4.00	5.00
2- reinforcement	103	2.12	0.73	1.00	1.67	2.00	2.67	3.67
3- intentions	103	4.09	0.62	2.67	3.67	4.00	4.67	5.00
4- beliefs about consequences	103	4.25	0.62	2.50	4.00	4.00	5.00	5.00
5- emotions	103	3.44	1.01	1.00	3.00	3.50	4.00	5.00
6- behavioural regulation	103	3.36	0.81	1.00	3.00	3.50	4.00	5.00

n: sample size; sd: standard deviation; min: minimum; Q0.25 first quartile; Q0.05: median; Q0.75: third quartile; max: maximum.

### Barriers and facilitators for self-harm prevention and surveillance

Notably, in preventing self-harm, knowledge and skills were predominantly a facilitator (58.1%), while context and institutional support were identified as barriers (75.7% and 91.7%, respectively).

Regarding self-harm surveillance, the domains “reinforcement” (89.8%) and “intentions” (56.55%) were identified as barriers, whilst the domains “knowledge and skills” (61.7%), “beliefs about consequences” (82.9%), “emotions” (55.8%), and “behavioural regulation” (61.4%) as facilitators (Fig 2).

### Associated factors to barriers and facilitators for self-harm prevention

The regression analysis showed that the independent variables ‘years since graduation’, ‘previous training in the topic’, ‘time working in PHC’, ‘having conducted self-harm prevention strategies’, and ‘working in a city with a self-harm prevention plan’, as well as working in a unit with a mental health-related standard operational procedure, were associated with the barriers and facilitators for self-harm prevention (Table 2).

**Table 2:** Associated factors for self-harm prevention according to regression analysis of data from PHC nurses.

Variables	Domain 1	Domain 2		Domain 3	
µ	β (SE), p	β (SE), p	AR [LI_AR, LS_AR]	β (SE), p	AR [LI_AR, LS_AR]
**(Intercept)**	2.9 (0.1), **0.00**	2.0 (0.0), **0.00**	–	1.3 (0.1), **0.00**	–
**Participant characteristics**					
Race (black/brown)	−0.3 (0.1), **0.01**	–	–	–	–
Years since graduation	–		–	0.0 (0.0), **0.01**	1.0 [1.0, 1.0]
Previous training around self-harm and suicide	0.5 (0.1), **0.00**	–	–	–	–
Time working in PHC	–	–	–	0.0 (0.0), **0.02**	1.0 [1.0, 1.0]
Has worked with a victim of suicide/self-harm before	0.4 (0.1), **0.00**	–	–	–	–
**Service characteristics**					
Presence of mental health specific agenda in the service	–	–		0.2 (0.1), **0.02**	1.3 [1.0, 1.5]
Presence of a local self-harm prevention strategy	0.5 (0.1), **0.00**	0.2 (0.1), **0.02**	1.2 [1.0, 1.4]	–	–
Presence of mental health-related operational protocol	–	–	–	0.4 (0.1), **0.01**	1.4 [1.1, 1.8]

Note: Domain 1- knowledge and skills, Domain 2 – context, Domain 3- institutional support [reinforcement]. µ: population mean of the dependent variable. β: regression coefficient estimate of the independent variable. SE: standard error of the estimate. P-value: significance level (<0.05). AR: adjusted odds ratio. LI_AR: Lower Interval of Adjusted Odds Ratio. LS_AR: Upper Interval of Adjusted Odds Ratio.

#### Knowledge and skills.

Scores in the “knowledge and skills” domain were higher among individuals with previous training, experience working with victims, and those working in a municipality with a prevention plan. Conversely, scores in the same domain were lower among black individuals. It was found that Black or Brown participants scored, on average, 0.27 points lower than white or yellow participants. Those who had training on the topic scored 0.48 points higher than those without training, and those who participated in prevention actions for victims scored 0.41 points higher in the same domain. Additionally, professionals working in cities with municipal prevention plans scored 0.53 points higher in this domain (Table 2).

#### Context.

According to Table 2, in the “context” domain, it was also identified that professionals working in cities with municipal prevention plans scored 18.80% higher compared to those working in towns without municipal plans.

#### Institutional support.

It was observed that for each additional year of education, the total score decreased by 2.53%, while each additional year of work in PHC increased the score by 2.37%. Professionals working in units with formal initiatives for mental health care scored 25.27% higher, and those in units with specific operational protocols for suicide prevention scored 42.42% higher in the institutional support domain.

### Associated factors to barriers and facilitators for self-harm surveillance

The regression analysis identified that the variables race (black/brown), female sex, years since graduation, previous training in the topic, having more than one employment positions, working in a place with matrix support, having worked with a victim of self-harm, knowing the self-harm notification form, and having experience in completing it were associated with the barriers and facilitators for the notification of self-harm (Table 3).

**Table 3:** Associated factors for self-harm surveillance, according to regression analysis of data from PHC nurses.

Variables	Domain 1	Domain 2		Domain 3		Domain 4	Domain 5	Domain 6	
µ	β (SE), p	β (SE), p	AR [LI_AR, LS_AR]	β (SE), p	AR [LI_AR, LS_AR]	β (SE), p	β ± SE, p	β (SE), p	AR [LI_AR, LS_AR
(Intercept)	2.9 (0.3), **0.00**	1.7 (0.2), **0.00**	–	2.3 (0.0), **0.00**	–	0.4 (0.1), **0.00**	2.0 ± 0.1, **0.00**	1.7 (0.1), **0.00**	–
**Participant characteristics**									
Race (Black/Brown)	−0.5 (0.1), **0.00**	–	–	–		–	–	–	–
Gender: female	−0.7 (0.2), **0.00**	–	–	–		–	–	0.2 (0.1), **0.03**	1.2 [1.0, 1.5]
Years since graduation	–	–	–	–		–	–	0.0 (0.0), **0.03**	1.0 [1.0, 1.0]
More than one income stream	0.6 (0.1), **0.00**	–	–	–		–	–	–	–
Time working in PHC	–	–	–	–		–	–	0.0 (0.0), **0.03**	1.0 [1.0, 1.0]
Has worked with a victim of suicide/self-harm before	–	−0.4 (0.2), **0.03**	0.7 [0.5, 0.9]	0.1 **± **0.0, **0.01**	1.1 [1.0, 1.2]	–	–	–	–
Has participated in prevention strategies for suicide/self-harm previously	–	–	–	0.1 (0.0), **0.00**	1.1 [1.0, 1.2]	−0.5 (0.3), 0.7	–	–	–
Has previous knowledge about the surveillance form	0.6 (0.1), **0.00**	–	–	–	–	–	–	–	–
Has previous experience with the surveillance form	0.7 (0.1), **0.00**	–	–	0.1 (0.0), **0.00**	1.1 [1.0, 1.2]	–	–	0.1 (0.0), **0.01**	1.1 [1.0, 1.2]
**Service characteristics**									
Presence of matrix support	0.3 (0.1), **0.00**	–	–	–		–	–	–	–
Presence of a local self-harm prevention strategy	0.4 (0.2), **0.04**	–	–	–	–	–	–	–	–
Presence of mental health-related operational protocol	–	–	–	–	–	–	–	0.2 (0.1), **0.03**	1.2 [1.0, 1.3]

Note: Domain 1 – Knowledge and skills, Domain 2 – Reinforcement, Domain 3 – Intentions, Domain 4 – Beliefs about Consequences, Domain 5 – Emotions, Domain 6 – Behavioural Regulation. µ: population mean of the dependent variable. β: regression coefficient estimate of the independent variable. SE: standard error of the estimate. pPvalue: significance level (<0.05). AR: adjusted odds ratio. LI_AR: Lower Interval of Adjusted Odds Ratio. LS_AR: Upper Interval of Adjusted Odds Ratio.

#### Knowledge and skills.

In the knowledge and skills domain, self-declared Black or Brown participants scored on average 0.52 points lower than self-declared White or Yellow participants. Female participants scored on average 0.72 points lower than male participants. Additionally, participants with multiple employment positions scored on average 0.64 points higher than those with only one employment position.

Participants working in a unit that was part of the matrix support strategy scored on average 0.35 points higher than those working in units that did not implement it. Participants who were familiar with the self-harm notification form and had previously completed it scored on average 0.63 and 0.74 points (respectively) higher than those who were not familiar with the form and had never completed it. Finally, participants working in a city with a municipal plan for preventing self-harm scored on average 0.38 points higher than those working in cities without such a plan.

#### Reinforcement.

Participants who had previously worked with victims of self-harm scored on average 32.8% lower in the reinforcement domain mean score compared to participants who had not worked with self-harm victims before.

#### Intentions.

In contrast, participants who had worked with victims of self-harm or had previously conducted prevention actions scored 13.65% and 9.83% higher, respectively, in the mean score of the ‘intentions’ domain compared to participants who had never worked with victims or conducted prevention actions for self-harm. Additionally, participants who had previously completed the surveillance form scored 9.49% higher on the same domain than participants who had never completed it.

#### Behavioural regulation.

In the behavioural regulation domain, female participants scored on average 22.54% higher than male participants. Additionally, participants who had completed the notification form scored 12.77% higher on the domain’s mean score than those who had never completed the form. Furthermore, participants working in a unit with a specific mental health operating protocol scored 17.27% higher on the mean behavioural regulation score than participants working in units without such a protocol.

Finally, for each additional year since graduation, the total score of the behavioural regulation domain for notification decreased by 1.04%. In contrast, for each additional year working in a PHC, the total behavioural regulation score increased by 1.06%.

## Discussion

To our knowledge, this is the first study that aimed to identify barriers and facilitators for self-harm prevention and surveillance in Brazil. Factors such as context, institutional support and reinforcement were identified as barriers. These factors should be considered by policymakers and managers when developing and implementing strategies for preventing and monitoring self-harm in the country. Although other studies have investigated the barriers and facilitators for self-harm and suicide prevention [14–16], the majority were conducted in high-income countries and those with smaller territorial extents, limiting the generalizability of the data to a country with the demographic, political, economic, social, and cultural characteristics of Brazil.

Factors related to knowledge and skills were identified as facilitators in both instruments (BFP and BFS). On the other hand, less than one-third of participants reported prior training or improvement in the studied topic. This finding raises questions about the importance of adopting a comprehensive approach to implementing prevention strategies through continuous education [11]. Several studies emphasise the importance of training primary health care (PHC) professionals to maximise the potential of this level of care in promoting mental health and preventing self-harm [1,31,32]. In Brazil, matrix support is recognised as a valuable strategy to address this issue, aiming to enhance mental health knowledge among PHC healthcare workers [33].

However, other evidence suggests that training and education alone are insufficient to modify practices in healthcare services, highlighting the need to examine individual, institutional, resource, and contextual barriers [34–35]. As shown by this study, factors related to beliefs about consequences, emotions, and behavioural regulation were also shown to be facilitators, especially for self-harm surveillance. Governmental efforts may influence these factors in developing legislation and documents that mandate self-harm surveillance as a public health measure, leading professionals to recognise the importance of monitoring and data in the development of policies and strategies [10].

Furthermore, the lack of institutional support and the work context were identified as barriers to self-harm prevention and surveillance. These findings align with recent international literature. Studies investigating the implementation of strategies for preventing self-harm have shown that the lack of organisational support and human resources act as significant barriers that must be addressed before implementing any prevention intervention [14–15]. Additionally, lack of time and resources acts as an important barrier for self-harm surveillance among young people and adolescents in high-income countries [11,14,16,36].

The fact that knowledge and skills scores were higher among professionals with previous training in the topic, prior experience in prevention strategies, and from cities with local prevention plans, highlights the importance of professional training and public efforts to develop actions that underpin clinical practice. Moreover, the fact that participants from cities with local prevention plans reported a greater perception of available resources further highlights the value of such documents in aligning strategies and deepening the understanding of self-harm within specific regional contexts [11,14,16].

The study found that Black and Brown individuals scored lower in the knowledge and skills domain. These data are relevant to the current panorama of the country and the findings of the present study. The latest survey by the Brazilian Nursing Council (COFEN) revealed that the majority of the nursing workforce in the country consists of individuals from black and brown backgrounds. It is known that the black and brown population in Brazil still has reduced access to education [37]. This could be related to the educational inequalities still present in Brazil. In the reality of Brazilian professionals, this may result in lower access to professional development courses, highlighting the importance of continuing education in healthcare services. A literature review found that several strategies, such as standardised interactive training, interdisciplinary approaches, stakeholder collaboration, distribution of educational materials, and post-training supervision and follow-up, can be effective in enhancing knowledge and skills for self-harm prevention [38].

It is worth noting that most participants reported lived experiences of self-harm. It is known that, depending on the type of experience and outcome, personal experience with self-harm can promote the reduction of stigma and negative feelings associated with the topic [39]. Additionally, experience working with victims of self-harm was shown to be a facilitator for the perception of knowledge and skills for prevention. In this regard, the literature suggests that prior contact with individuals who have lived experience can contribute to more effective and compassionate care, as education through contact can help reduce stigma in mental health [40].

This study found that the majority of participants reported an absence of a dedicated mental health care agenda or self-harm prevention protocols in their workplaces. A lack of organisational structure and support emerged as a significant barrier to self-harm prevention. Perceived institutional support was lower among those with higher levels of education, but higher among individuals with more years of experience in PHC, those working in units with formal mental health initiatives, and those in units with specific protocols for self-harm prevention. These findings highlight the importance of having context-specific, structured protocols, strategies, and preventive measures that reflect the realities faced by healthcare professionals and the public [41]. A systematic review showed that insubstantial mental health policies and taboos limit research and academic publication in suicidality in Brazil, which can impact the dissemination of information [42].

The WHO emphasises that suicide prevention is generally not a priority for policymakers and that a robust national prevention policy is necessary for commitment and prioritisation of the agenda [21]. Brazil was a pioneer in implementing a National Suicide Prevention Policy in Latin America in 2006. However, the implementation of this policy has still fallen short of expectations, and there is a lack of studies investigating this issue. It is known that the country still faces organisational and political challenges, such as a lack of intersectoral coordination and insufficient integration with primary care practices, which impact the implementation of the national policy [42]. While this study did not directly evaluate public policies, the identification of key barriers and facilitators suggests areas where policies and strategies could be strengthened to support self-harm prevention and surveillance in primary care.

## Conclusion

In conclusion, this study provides valuable insights into the barriers and facilitators of self-harm prevention in Brazil. Barriers were associated with institutional and contextual factors, whereas facilitators were more closely related to individual characteristics. By identifying key barriers, such as limited institutional support and the lack of structured protocols and resources, the study highlights the need for more well-resourced, context-sensitive strategies, as well as the importance of engaging policymakers and institutions. These findings highlight the need for further examination of how national and local regulations are implemented in practice, and how they can be strengthened to better support professionals and services. We emphasise that Brazil is a vast country with significant cultural and political diversity, and that careful consideration should be given to each of its regions. One limitation of this study is that the final sample size was smaller than the initially calculated sample size. Recruitment challenges, including non-response, prevented full achievement of the target sample. As a result, the statistical power to detect smaller effects may have been reduced. Nevertheless, the analysis was conducted using robust modelling techniques (GAMLSS), and the findings remain informative for understanding the factors associated with prevention and surveillance practices in primary care in Brazil. Therefore, future studies should focus on investigating barriers and facilitators using larger samples, exploring the potential of more context-specific implementation strategies.
